# The Removal of Cr(VI) from Aqueous Solutions with Corn Stalk Biochar

**DOI:** 10.3390/ijerph192114188

**Published:** 2022-10-30

**Authors:** Wenling Yang, Gao Lei, Shujing Quan, Longfei Zhang, Baitao Wang, Hong Hu, Liangliang Li, Huan Ma, Chaohui Yin, Fei Feng, Yanyan Jing

**Affiliations:** 1 Key Laboratory of Microbial Engineering at the Institute of Biology, Henan Academy of Sciences, Zhengzhou 450008, China; 2 Key Laboratory of New Materials and Facilities for Rural Renewable Energy (Ministry of Agriculture and Rural Affairs of China), Henan Agricultural University, Zhengzhou 450002, China

**Keywords:** adsorption ability, biochar, Cr(VI), optimization, removal efficiency

## Abstract

The discharge of wastewater containing hexavalent chromium (Cr(VI)) into the environment is very harmful to living things. Therefore, before effluent that contains Cr(VI) can be discharged into the environment, this toxin should be removed from the contaminated water. In this study, corn stalk biochar was investigated to evaluate the Cr(VI) removal efficiency from an aqueous solution. The effects of pH (2–10), biochar concentration (0.5 to 10 g/L), Cr(VI) concentration (10–500 mg/L), and contact time (10–1440 min) were studied. The actual experimental value of the Cr(VI) removal efficiency was 28.67%, largely consistent with the predicted model value of 29.31%, under the optimal conditions of a Cr(VI) concentration of 60 g/L, pH 4, contact time of 270 min, and a biochar concentration of 4.5 g/L. A significant interaction between the Cr(VI) concentration and pH was observed, along with significance in the interaction between Cr(VI) concentration and biochar concentration, which had a greater impact on the removal of Cr(VI). Biosorption onto corn stalk biochar is an affordable and economical adsorption process to treat wastewater contaminated with Cr(VI). The aim of this study is to provide data to serve as a basis for future studies on the use of raw agricultural waste to remove Cr(VI).

## 1. Introduction

Chromium (Cr) is considered a very toxic environmental contaminant. Hexavalent chromium (Cr(VI)) is highly toxic, non-degradable, soluble, mobile, concealed, and can have an adverse impact on ecological sustainability and human health [1,2,3]. Therefore, before effluent is discharged into the environment, Cr(VI) should be removed from the contaminated water.

Currently, the methods that are commonly used to remove Cr from wastewater primarily include ion exchange, chemical precipitation, electrochemistry, membrane filtration, and adsorption, among others [4,5,6,7,8]. Among these methods, adsorption is considered a suitable method to remove Cr in wastewater owing to its advantages of convenient operation and environmental protection [7,9,10,11,12,13,14]. However, the high cost of adsorbents limits their application in heavy metal wastewater treatment. Therefore, the key to the success of the adsorption method is to find environmentally friendly, inexpensive, and efficient adsorption materials.

In recent years, biomass raw materials represented by straw, livestock manure, and industrial sludge have attracted extensive attention. Most of these raw biomass materials originate from byproducts or wastes in industrial and agricultural production processes, with wide sources and low cost, and have enormous application prospects in the field of adsorption [8,15,16,17,18,19]. However, the direct use of biomass feedstocks as adsorbents is inefficient [4]. The use of biochar produced by the pyrolysis of agro-industrial by-products for the adsorption of heavy metals in wastewater can not only turn waste into treasure but also greatly improve the adsorption efficiency of heavy metals in wastewater, while reducing its harm to the environment.

Biochar is an alkaline, carbon-based material with a large specific surface area and porosity, high aromatic structure, surface functional groups, such as carboxyl, hydroxyl, and acid anhydride, and other surface physicochemical properties. It is effective at adsorbing Cr in wastewater [7,8,13]. However, there are some factors that affect the removal efficiency of Cr(VI) by biochar. The pyrolysis temperature of biochar and the optimal environmental conditions for Cr(VI) removal by biochar all affect the removal efficiency of Cr(VI) in practical applications. The reduction of Cr(VI) by biochar derived from tobacco petiole pyrolysis was primarily responsible for carboxyl that was highly dependent on aqueous pH, and the removal efficiency of tobacco petiole biochar on Cr(VI) was inversely proportional to the pyrolytic temperature (PyT), which affected functional groups rather than a specific surface area [7]. Chaenomeles sinensis seed (CSS) biochar prepared at 450 °C had increased adsorption capacity for Cr(VI) and Cu(II) compared with that prepared at 300 °C and 600 °C, which were 93.19 mg/g and 105.12 mg/g, respectively [12]. Studies have shown that acidic conditions aid in the removal of Cr(VI) by biochar. For example, the remediation of Cr(VI) using walnut shell biochar was proven to be effective and removed the maximum concentration of Cr(VI), up to 93%, at pH 5.5, 2 h agitation time, and 1.1 g/L biochar from an aqueous solution [13]. The biochar prepared from different garden wastes can effectively remove Cr(VI) under acidic conditions, and the highest removal efficiency was observed at pH 2 [8]. However, the removal of Cr(VI) by CSS biochar was highly efficient over a wide range of pH values (1–10), whereas the removal of Cu(II) was pH dependent, with an optimum at pH = 6 [12].

**Figure 1:**
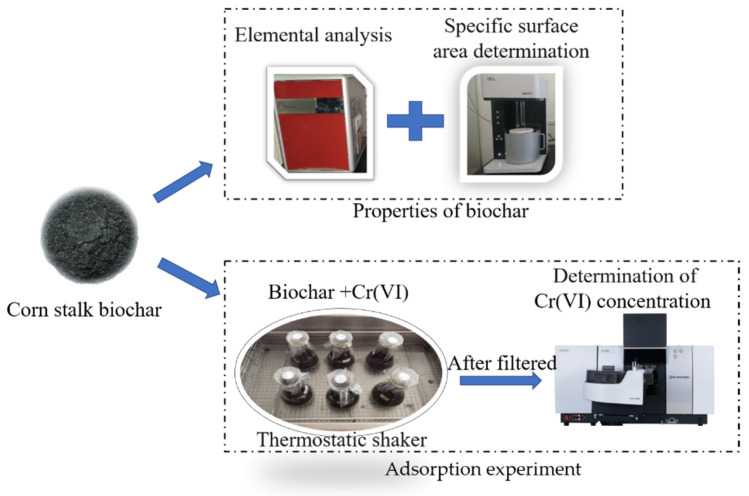
The schematic illustration of adsorption experiments.

Although studies have been conducted on the production of biochar using these agro-industrial byproducts, it is important to consider the actual application conditions that would facilitate their preparation for batch adsorption studies. Response surface methodology (RSM) is a statistical method which uses multiple quadratic regression equations to fit the functional relationship between factors and response values and seeks the optimal parameters through the analysis of regression equations, so as to solve multivariable problems. Box–Behnken design (BBD) is one of the commonly used response surface analyses, which is mainly applied to the optimization of process variables with less than five factors and three levels [20,21,22,23]. Moreover, the BBD has the advantages of being able to evaluate the nonlinear influence of factors and requiring fewer tests than the central composite design (CCD) when the number of factors is the same. Therefore, taking corn stalk biochar as adsorbent, this paper investigated the effects of pH, biochar concentration, Cr(VI) concentration, and contact time on Cr(VI) removal (adsorption experiments as Figure 1), evaluated the interaction of factors, and analyzed the optimal process conditions for Cr(VI) removal using the BBD response surface method.

## 2. Materials and Methods

### 2.1. Elemental Composition of Biochar

The elemental composition of corn stalk biochar (80 mesh) was determined by a Vario EL Cube elemental analyzer. The air pressure of carrier gases was 0.20 MPa for O_2_ and 0.12 MPa for He.

### 2.2. Specific Surface Area of Biochar

The specific surface area of biochar was determined via a BELSORP-mini Ⅱ instrument using N_2_ and He as carrier gas at 103 KPa. The specific surface area test specification was greater than 0.01 m^2^/g.

### 2.3. Preparation of Cr(VI) Solutions

A Cr(VI) stock solution (500 mg/L) was prepared using K_2_Cr_2_O_7_. The Cr(VI) working solutions were obtained by diluting the Cr(VI) stock solution. The absorbance values were taken from an atomic absorption spectrophotometer (AA-6880F/AAC, Shimadzu, Japan) with a wavelength accuracy of ±0.3 nm. The Cr(VI) concentration standard curve (1) was generated.

y = 0.0343x + 0.0074 R^2^ = 0.9927    (1)

### 2.4. Adsorption Experimental Design

The adsorption experiments were conducted in triangular bottles. Working solutions of Cr(VI) and biochar were added to each triangular bottle, which was then sealed and placed in a thermostatic shaker maintained at 25 ± 2 °C and operated at a mixing speed of 150 rpm. The effect of the four main parameters, the concentration of biochar (0.5 to 10 g/L), pH (2 to 10), contact time (10 to 1440 min), and the concentration of Cr(VI) (10–500 mg/L), on the adsorption capacity of biochar were investigated. To adjust the pH of solutions, 0.1 mol/L solutions of HCl and NaOH were used, and pH values were determined using a pH meter (FE28, METTLER TOLEDO, Zurich, Switzerland).

After the adsorption experiments, samples were filtered through a syringe filter (0.45 μm), and residual concentrations of Cr(VI) in solution were measured by an AA-6880F/AAC atomic absorption spectrophotometer (Shimadzu, Japan) with a wavelength accuracy of ±0.3 nm. The instrument was operated under an output pressure of 0.35 MPa of an air compressor and an outlet pressure of 0.09 MPa. The pressure of the carrier gas (C_2_H_2_) was greater than 0.5 MPa.

The adsorption amount (mg/g) of Cr(VI) by biochar was determined using Equation (2), and the removal efficiency (%) of Cr(VI) by biochar was determined using Equation (3) [24].

(2) $$Q=\frac{\left(C_{0}-C_{1}\right)V}{m}$$

(3) $$R=\frac{C_{0}-C_{1}}{C_{0}}\times 100$$

where *C*_0_ and *C*_1_ are the measured Cr(VI) concentrations (mg/L) before adsorption and after a specific time of adsorption (*t*, min); *V* is the volume of the Cr(VI)-containing aqueous solution, and *m* is the mass of biochar (g).

### 2.5. Response Surface Design

The Box–Behnken design (BBD) response surface method was designed to optimize the experiment to obtain the optimal conditions for Cr(VI) removal by corn straw biochar. In the experiment, the removal efficiency (%) of Cr(VI) by biochar was used as the evaluation index and four factors, the Cr(VI) concentration (40 mg/L, 60 mg/L, and 80 mg/L), pH (2, 4, and 6), biochar concentration (2 g/L, 4 g/L, and 6 g/L), and contact time (120 min, 240 min, and 360 min) were established. Each factor had three levels. The experimental design scheme is shown in Table 1.

## 3. Results and Discussions

### 3.1. The Properties of Biochar

The corn straw biochar was primarily composed of C, H, N, and S, with the highest carbon content of 49.15% and the lowest sulfur content of 0.16%, in which the carbon hydrogen ratio was 27.20 and the carbon nitrogen ratio was as high as 52.16, which was very different from the elemental composition of straw biomass (carbon hydrogen ratio 7.44 and carbon nitrogen ratio 24.26). The total pore volume of biochar was 1.71 × 10^−2^ cm^3^/g, the specific surface area was 3.36 m^2^/g, and the average pore diameter was 20.32 nm.

### 3.2. Effect of Biochar Concentration on Cr(VI) Removal

**Figure 2:**
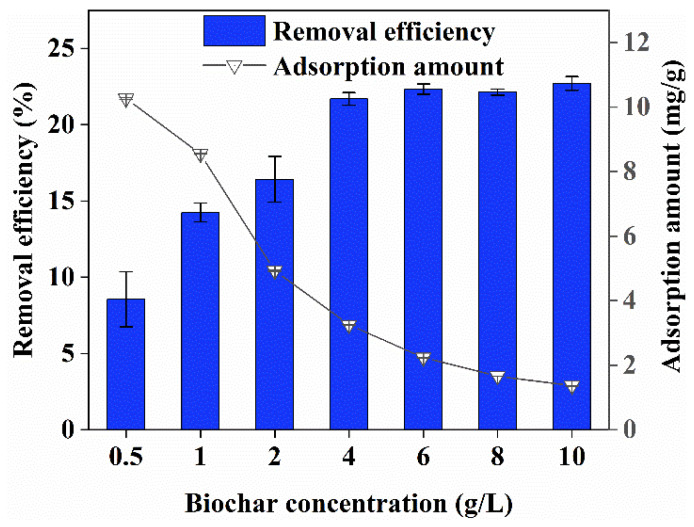
Effect of biochar concentration on Cr(VI) removal.

The effect of biochar concentration on the adsorption of Cr(VI) is shown in Figure 2. This experiment was conducted at a pH of 7, Cr(VI) concentration of 100 mg/L, contact time of 1440 min, mixing speed of 150 rpm, and temperature of 25 ± 2 °C. The concentrations of biochar were 0.5, 1, 2, 4, 6, 8, and 10 g/L.

With the increase in biochar concentration, the Cr(VI) removal efficiency by biochar increased, and the adsorption amount decreased as the concentration of biochar increased. This was because the adsorption sites on Cr(VI) in the solution increased in parallel with the concentration of biochar; therefore, the total number of active sites increased, resulting in easier adsorption of Cr(VI), with a concomitant increase in removal efficiency [11]. However, as the concentration of Cr(VI) in the solution was fixed, there were surplus adsorption sites of biochar, and there was excess relative to Cr(VI), resulting in the unsaturation of Cr(VI) adsorption by the unit mass biochar. Thus, the adsorption capacity of the unit mass biochar was reduced. When the concentration of biochar was 4 g/L, the Cr(VI) removal efficiency was 21.69%, and the adsorption amount was 3.25 mg/g. With the continuous increase in the concentration of biochar, although the Cr(VI) removal efficiency still increased, the amount of adsorption decreased. The Cr(VI) removal efficiency by biochar only increased by 1.01% with biochar concentrations that ranged from 4 g/L to 10 g/L. This was because the Cr(VI) concentration limited the increase in adsorption efficiency. In conclusion, 4 g/L was a better concentration.

### 3.3. Effect of Contact Time on Cr(VI) Removal

**Figure 3:**
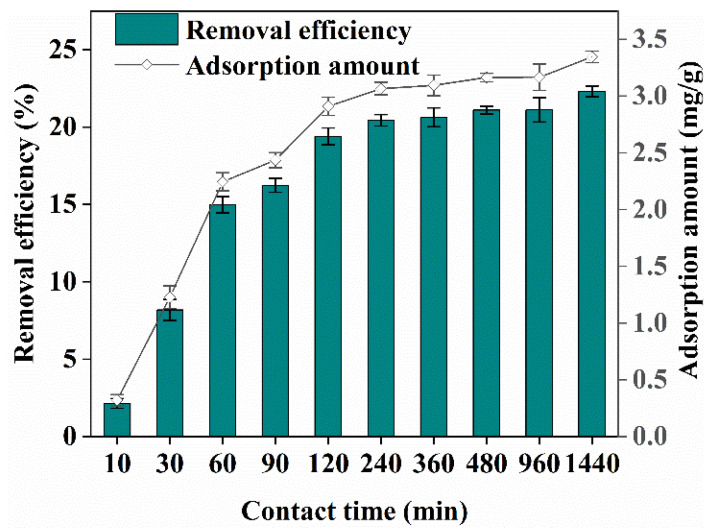
Effect of contact time on Cr(VI) removal.

The effect of contact time on Cr(VI) removal when the concentration of Cr(VI) in the solution was 100 mg/L, the pH was 7, the biochar concentration was 4 g/L, and the contact times were 10, 30, 60, 90, 120, 240, 360, 480, 960, and 1440 min, is shown in Figure 3. The surface adsorption and functional group adsorption increased the reaction rate during the first 0–90 min owing to many unsaturated adsorption sites on the surface of the biochar. Thus, the adsorption amount and Cr(VI) removal efficiency by biochar increased rapidly with the increase in contact time. Within the 120–360 min range, since most of the chromium ions have been adsorbed by the biochar, the difference in Cr(VI) concentration between the biochar and the solid–liquid phase of the solution decreased, resulting in a decrease in the mass transfer power in the solution, and then the increase of adsorption amount and removal efficiency was obviously slowed down [11,21]. The removal efficiency and adsorption amount of Cr(VI) by biochar reached 20.44% and 3.07 mg/g, respectively, at 240 min. After this time, the adsorption capacity remained constant or changed slightly. The maximum Cr(VI) removal efficiency and adsorption amount at 1440 min were 22.30% and 3.35 mg/g, respectively. Before the adsorption equilibrium was reached, sufficient contact time favored the adsorption and Cr(VI) removal by biochar. Similar findings were reported by Harifi-Mood et al. [11].

### 3.4. Effect of Cr(VI) Concentration on Cr(VI) Removal

**Figure 4:**
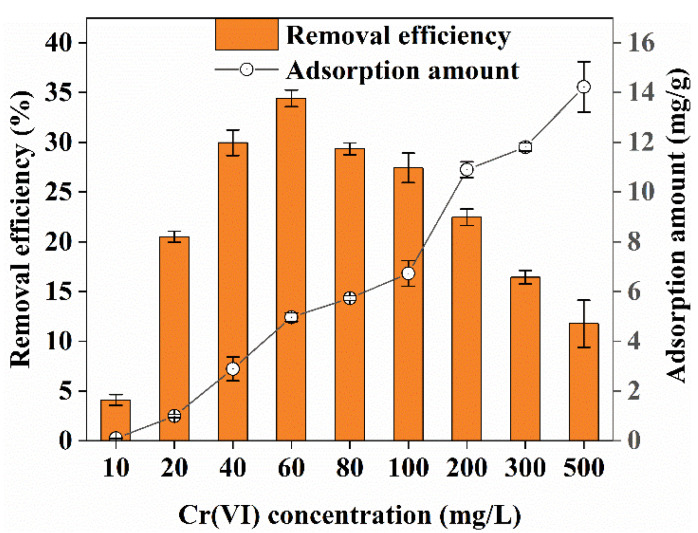
Effect of Cr(VI) concentration on Cr(VI) removal.

The effect of Cr(VI) concentration on adsorption when the biochar concentration was 4 g/L and the contact time was 360 min is shown in Figure 4. The Cr(VI) removal efficiency by biochar showed a trend of first increasing and then decreasing, whereas the adsorption amount continued to increase. When the Cr(VI) concentration was 60 mg/L, the Cr(VI) removal efficiency reached a maximum of 34.42%. The adsorption amount was 4.96 mg/g. In contrast, when the Cr(VI) concentration in solution was 500 mg/L, the maximum adsorption amount was 14.23 mg/g and the Cr(VI) removal efficiency was only 11.76%. When the Cr(VI) concentration in the solution was lower than 60 mg/L, the Cr(VI) removal efficiency and adsorption amount continued to increase. However, when the Cr(VI) concentration was higher than 60 mg/L, the Cr(VI) removal efficiency began to decrease, whereas the amount of adsorption continued to increase. This was because when the concentration of Cr(VI) was less than 60 mg/L, the adsorption sites of biochar were relatively sufficient and could absorb more strongly. As the concentration of Cr(VI) increased, increasing amounts of Cr(VI) were in contact with the surface of the biochar, resulting in larger adsorption efficiency and amounts absorbed. While the adsorbent had a limited number of adsorption sites and when the concentration of Cr(VI) in the aqueous solution increased, the competition to occupy the available adsorption locations by Cr(VI) intensified. Thus, the removal efficiency decreased [25]. When the concentration of Cr(VI) was 40 mg/L and 80 mg/L, the removal efficiency was 29.95% and 29.35%, respectively, which was approximately 5% different from the maximum adsorption efficiency of 34.42%. Therefore, an initial concentration of 40–80 mg/L Cr(VI) could be taken as the best value in the adsorption system using the corn straw biochar prepared in this study.

### 3.5. Effect of pH on Cr(VI) Removal

**Figure 5:**
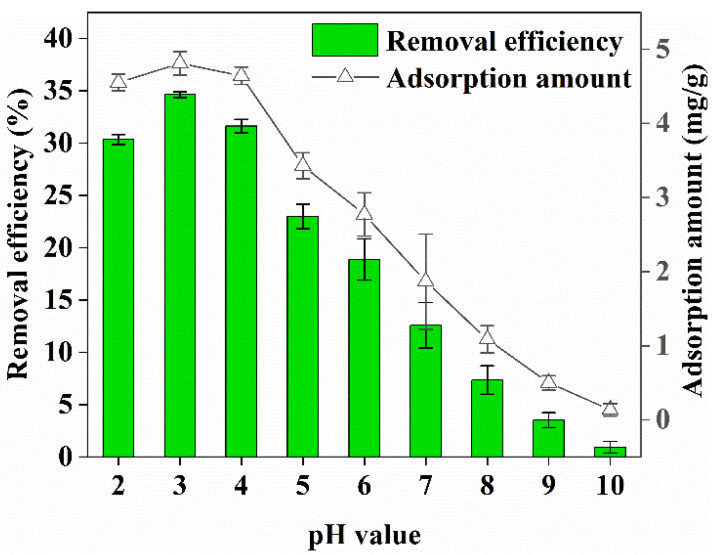
Effect of pH on Cr(VI) removal.

The effect of pH values in the range 2–10 on Cr(VI) adsorption by biochar when the condition of Cr(VI) concentration in the solution was 60 mg/L, that of biochar was 4 g/L, and contact time was 360 min is shown in Figure 5. The biochar was highly efficient at removing Cr(VI) from the wastewater at pH 2–4, and the Cr(VI) removal efficiency and adsorption amount reached their maximum values of 34.64% and 4.81 mg/g, respectively, at pH 3.0.

At pH 5–6, the adsorption amount and removal efficiency of biochar for Cr(VI) in wastewater gradually decreased, but the Cr(VI) removal efficiency was above 18%. When the pH value was greater than 7, the biochar was no longer suitable for the removal of Cr(VI). The Cr(VI) removal efficiency and adsorption amounts were 0.93% and 0.13 mg/g, respectively, at pH 10. This was because the structure of oxygen-containing functional groups in the biochar was highly stable and active under acidic conditions and they could strongly absorb Cr(VI). However, the structure of the oxygen-containing functional groups was continuously destroyed as the pH increased, particularly when the pH was greater than 7. The heavy metal ions were hydrolyzed to form their corresponding hydroxides that were then deposited on the surface of the biochar; this affected the active sites of biosorption. The current results are consistent with those obtained by Harifi-Mood et al. [11], who studied the removal of Cr(VI) using almond green hull power. Similarly, in a study on Cr(VI) adsorption from aqueous solution by powdered activated carbon synthesized from Peganum harmala seeds by ultrasonic wave activation, Nasseh et al. [25] observed that the highest removal efficiency (99.6%) was obtained at pH 3, and the removal efficiency decreased below and above this pH value.

### 3.6. Optimization of Cr(VI) Adsorption Process by Biochar

According to the univariate test results and the scheme of the Box–Behnken experimental design in Table 1, a multiple regression analysis was performed on the experimental data. As a function of variables *A* (Cr[VI] concentration), *B* (pH), *C* (biochar concentration), and *D* (contact time), the quadratic regression model equations for coded values (*X*_Coded_) and actual experimental values (*X*_Actual_) were given as Equation (4) and Equation (5), respectively. Table 2 displays the response in different experimental runs.

(4) $$\begin{array}{l} X_{C\mathrm{oded}}=28.68+0.0367A-0.47B+2.9C+1.69D-0.7725AB+0.265AC\\ +0.0875AD-0.1525BC+0.015BD-0.0075CD-2.41A^{2}\\ -4.51B^{2}-5.22C^{2}-3.48D^{2} \end{array}$$

(5) $$\begin{array}{l} X_{Actual}=-57.30917+0.766583A+10.08458B+11.64583C+0.127937D\\ -0.019313AB+0.006625AC+0.000036AD-0.038125BC\\ +0.000062BD-0.000031CD-0.006023A^{2}-1.12792B^{2}\\ -1.30417C^{2}-0.000242D^{2} \end{array}$$

Table 3 shows the analysis of variance (ANOVA) of the model.

The fitting degree of the regression equation to the data depends on the determination coefficient *R*^2^. The closer *R*^2^ is to 1, the more accurate the fitted equation and the better the fitting degree. The determination coefficient *R*^2^ of the fitted model was 0.9986, and the adjusted *R*^2^ was 0.9972, which was relatively close to 1, indicating that this model could explain 99.72% of the response value variation. There was no significant difference in the *p*-value of the lack of fit (0.4204 > 0.05), indicating that the model fitted the experimental data well. The factor *p*-value of <0.05 indicated that this factor has a significant impact on the model. In this model, pH (*B*), biochar concentration (*C*), and contact time (*D*) all had significant effects on the Cr(VI) absorption by biochar (*p <* 0.05). In the quadratic model terms, the interaction between Cr(VI) concentration and pH (*AB*) had a significant effect on Cr(VI) removal, along with the significance in the interaction between the concentrations of Cr(VI) and biochar (*AC*). Simultaneously, it is apparent from this optimized experiment that the Cr(VI) removal efficiency was the highest and the predicted value was 29.31% under the conditions of a concentration of Cr(VI) of 60 g/L, a pH of 4, a contact time of 270 min, and a biochar concentration of 4.5 g/L.

**Figure 6:**
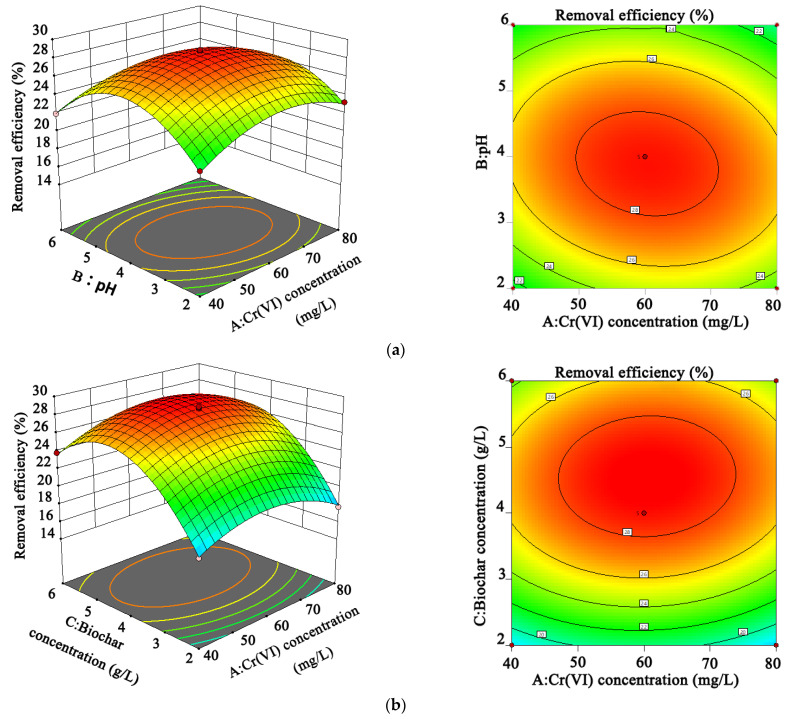

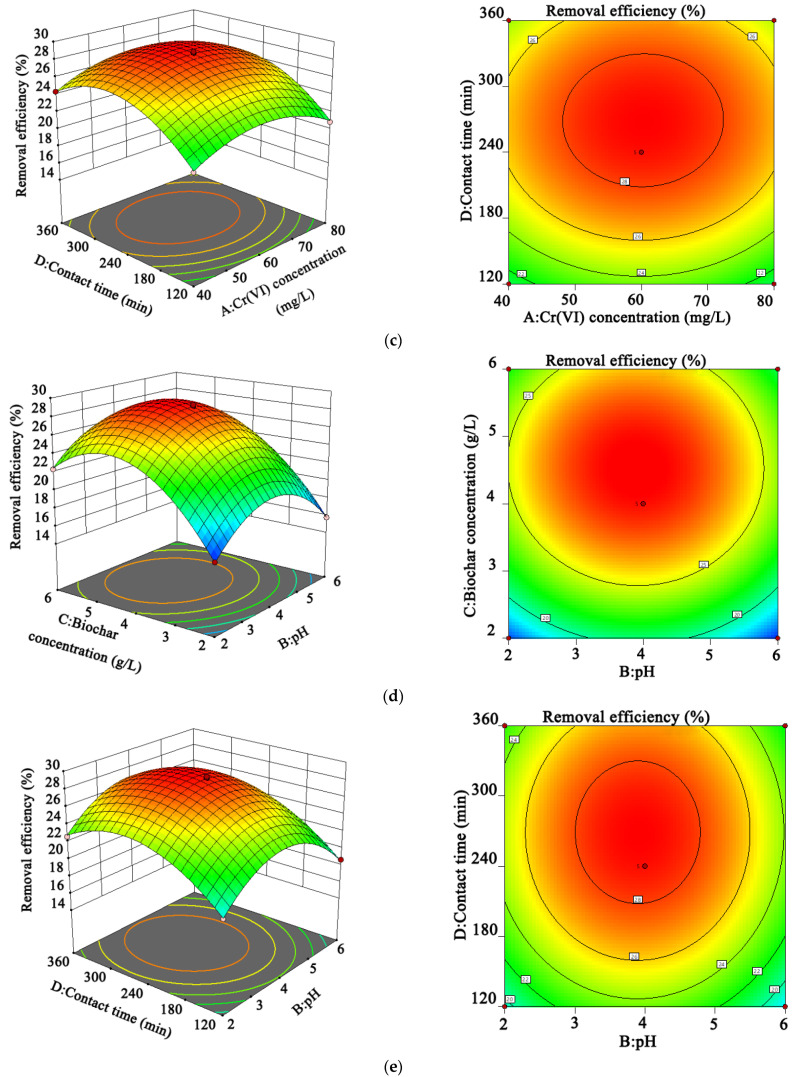

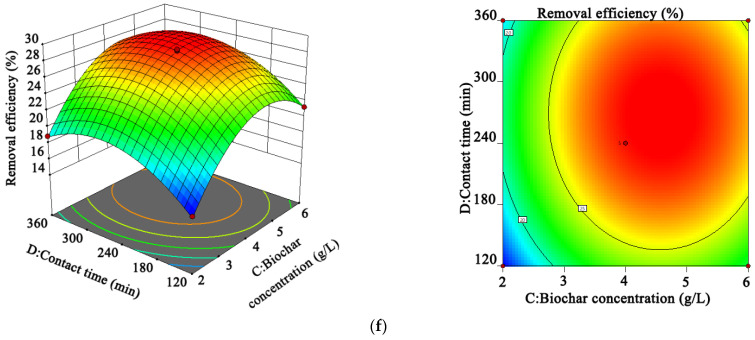
Contour plot and 3-D surface plot for Cr (VI) removal. (**a**) interaction between the Cr(VI) concentration and pH; (**b**) interaction between the Cr(VI) and biochar concentration; (**c**) interaction between Cr(VI) concentration and contact time; (**d**) interaction between pH and biochar concentration; (**e**) interaction between pH and contact time; (**f**) interaction between biochar concentration and contact time.

Figure 6 shows a three-dimensional (3-D) response surface and contour plot of the interaction of each factor. The response surface of the model was drawn by keeping two variables constant within the experimental range and varying the other two variables. The color and shape of the contour plots identifies significance and interactions between the variables. The interaction between the Cr(VI) concentration and pH (*AB*) had a significant effect on Cr(VI) removal (*p* < 0.05), along with the significance in the interaction between the Cr(VI) and biochar concentrations (*AC*) (Figure 6a,b). This conclusion was consistent with the ANOVA results: *p*-value of *AB* (*p <* 0.0001) and *p*-value of *AC* (*p* = 0.0235).

The 3-D response surface and contour plots of Cr(VI) removal with Cr(VI) concentration (*A*) and pH value (*B*) under the conditions of a constant concentration of biochar (4 g/L) and adsorption contact time (280 min) are shown in Figure 6a. With the increase in the concentration of Cr(VI) in solution and of the pH value, the adsorption capacity of biochar to Cr(VI) showed a trend to first increase and then decrease, which was related to the number of adsorption sites on the surface of biochar and the solid–liquid two-phase mass transfer resistance. The change in ligand exchange between the chromium ions and biochar functional groups owing to the pH value affected the adsorption performance of the biochar. Figure 6b shows the change in Cr(VI) removal efficiency with the concentrations of Cr(VI) (*A*) and biochar (*C*) were changing and the pH value and adsorption time were constant. When the biochar concentration increased, the Cr(VI) adsorption sites in the solution increased, the Cr(VI) removal efficiency increased, and the adsorption amount per unit mass of biochar decreased. Simultaneously, the Cr(VI) adsorption by biochar was related to the balance between adsorption and desorption in the system. Excessive concentrations of Cr(VI) in the solution resulted in a decrease of Cr(VI) removal efficiency. There was no significant interaction between the two variables in Figure 6c–f where their ANOVA results had *p*-values >0.05 (Table 3).

**Figure 7:**
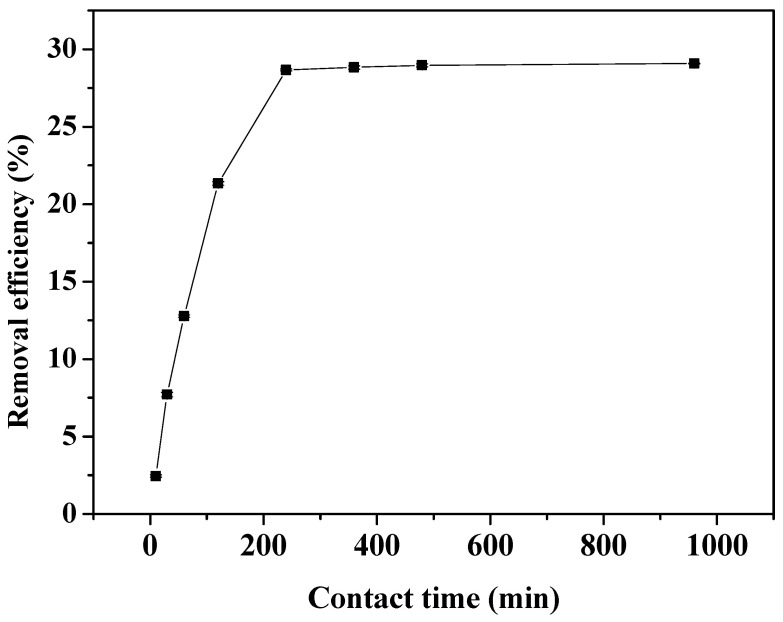
The removal efficiency under the optimal conditions.

To verify the adsorption effect of biochar on Cr(VI) predicted by the model, the adsorption experiment was conducted under the optimal conditions of a Cr(VI) concentration of 60 g/L, a pH of 4, a contact time of 270 min, and a biochar concentration of 4.5 g/L. The actual experimental value of Cr(VI) removal efficiency was 28.67% (Figure 7), which was largely consistent with the model prediction value of 29.31% and proving that the simulation results were accurate and feasible.

## 4. Conclusions

Corn straw biochar shows potential for environmental applications to decontaminate wastewater contaminated with Cr(VI). Acidic conditions favor the removal of Cr(VI) by biochar. As the concentration of Cr(VI) increased in solution and the pH value increased, the adsorption capacity of biochar for Cr(VI) showed a trend of first increasing and then decreasing. The Cr(VI) removal efficiency by biochar increased and the adsorption amount decreased as the concentration of biochar increased. The interaction between Cr(VI) concentration and pH had a significant effect on Cr(VI) removal, as did the interaction between the concentrations of Cr(VI) and biochar. The actual experimental value of Cr(VI) removal efficiency was 28.67%, which was largely consistent with the model prediction value of 29.31% based on the optimal conditions of a Cr(VI) concentration of 60 g/L, a pH value of 4, a contact time of 270 min, and a biochar concentration of 4.5 g/L.

## Figures and Tables

**Table 1:** BBD response surface test design table.

Code	Variables	Unit	Low Level (−1)	High Level (+1)
*A*	Cr(VI) concentration	mg/L	40	80
*B*	pH		2	6
*C*	Biochar concentration	(g/L)	2	6
*D*	Contact time	(min)	120	360

**Table 2:** The coded values for independent factors and the values of measured response.

Code	Cr(VI) Concentration (mg/L)		pH		Biochar Concentration (g/L)		Contact Time (min)		R (%)
	*A*	Coded	*B*	Coded	*C*	Coded	*D*	Coded	
1	60	0	6	+1	2	−1	240	0	15.72
2	60	0	4	0	4	0	240	0	28.69
3	80	+1	4	0	6	+1	240	0	24.38
4	60	0	4	0	6	+1	360	+1	24.55
5	60	0	4	0	4	0	240	0	28.75
6	80	+1	6	+1	4	0	240	+1	20.58
7	60	0	6	+1	4	0	360	+1	21.99
8	60	0	2	−1	4	0	360	+1	22.65
9	40	−1	4	0	4	0	120	−1	20.98
10	60	0	2	−1	2	−1	240	0	16.42
11	60	0	6	+1	6	+1	240	0	21.02
12	60	0	4	0	4	0	240	0	28.31
13	60	0	2	−1	6	+1	240	0	22.33
14	40	−1	6	+1	4	0	240	0	21.98
15	80	+1	4	0	2	−1	240	0	17.73
16	60	0	4	0	4	0	240	0	28.78
17	40	−1	4	0	4	0	360	+1	24.37
18	60	0	2	−1	4	0	120	−1	19.40
19	60	0	4	0	2	−1	120	−1	15.56
20	80	+1	4	0	4	0	360	+1	24.61
21	60	0	4	0	6	+1	120	−1	21.25
22	60	0	4	0	4	0	240	0	28.87
23	80	+1	4	0	4	0	120	−1	20.87
24	60	0	6	+1	4	0	120	−1	18.68
25	40	−1	2	−1	4	0	240	0	21.56
26	80	+1	2	−1	4	0	240	0	23.25
27	60	0	4	0	2	−1	360	+1	18.89
28	40	−1	4	0	6	+1	240	0	23.84
29	40	−1	4	0	2	−1	240	0	18.25

**Table 3:** Analysis of variance of the model.

Source	Sum of Squares	Degree Freedom	Mean Square	F-Value	*p*-Value
Model	429.26	14	30.66	705.60	<0.0001
*A*	0.0161	1	0.0161	0.3713	0.5521
*B*	2.65	1	2.65	61.00	<0.0001
*C*	100.92	1	100.92	2322.42	<0.0001
*D*	34.41	1	34.41	791.82	<0.0001
*AB*	2.39	1	2.39	54.93	<0.0001
*AC*	0.2809	1	0.2809	6.46	0.0235
*AD*	0.0306	1	0.0306	0.7048	0.4153
*BC*	0.0930	1	0.0930	2.14	0.1655
*BD*	0.0009	1	0.0009	0.0207	0.8876
*CD*	0.0002	1	0.0002	0.0052	0.9437
*A* ^2^	37.65	1	37.65	866.37	<0.0001
*B* ^2^	132.03	1	132.03	3038.41	<0.0001
*C* ^2^	176.52	1	176.52	4062.17	<0.0001
*D* ^2^	78.74	1	78.74	1812.05	<0.0001
Residual	0.6084	14	0.0435		
Lack of fit	0.4204	10	0.0420	0.8944	0.5991
Pure error	0.1880	4	0.0470		
Cor total	429.87	28			

*R*^2^ = 0.9986 adjusted *R*^2^ = 0.9972.

## Data Availability

All relevant data are included in the paper.
